# The Influence of TiO_2_ Nanoparticles Morphologies on the Performance of Lithium-Ion Batteries

**DOI:** 10.3390/nano13192636

**Published:** 2023-09-25

**Authors:** Wenpo Luo, Juliette Blanchard, Yanpeng Xue, Abdelhafed Taleb

**Affiliations:** 1 Chimie ParisTech—CNRS, Institut de Recherche de Chimie Paris, PSL Research University, 75005 Paris, France; wenpo.luo@chimieparistech.psl.eu; 2 Laboratoire de Réactivité de Surface (LRS), Sorbonne Université, 4 Place Jussieu, 75231 Paris, France; juliette.blanchard@sorbonne-universite.fr; 3 National Center for Materials Service Safety, University of Science and Technology Beijing, Xueyuan Road 30, Beijing 100083, China; xueyanpeng789@163.com; 4 Sorbonne Université, 4 Place Jussieu, 75231 Paris, France

**Keywords:** Titania, nanoparticles, aggregates, morphologies, Li-ion batteries

## Abstract

Anode materials based on the TiO_2_ nanoparticles of different morphologies were prepared using the hydrothermal method and characterized by various techniques, such as X-ray diffraction (XRD), field-emission scanning electron microscopy (FE-SEM), and N_2_ absorption. The TiO_2_ nanoparticles prepared were used as anode materials for lithium-ion batteries (LIBs), and their electrochemical properties were tested using discharging/charging measurements. The results showed that the initial morphology of the nanoparticles plays a minor role in battery performance after the first few cycles and that better capacity was achieved for TiO_2_ nanobelt morphology. The sharp drop in the specific capacity of LIB during their first cycles is examined by considering changes in the morphology of TiO_2_ particles and their porosity properties in terms of size and connectivity. The performance of TiO_2_ anode materials has also been assessed by considering their phase.

## 1. Introduction

Renewable energies are increasingly becoming an alternative solution for overcoming the problems of fossil fuel pollution and meeting the growing demand for energy from human activity. However, to overcome the problem of intermittency, which limits their use when needed, renewable energy storage systems are currently emerging as credible and effective solutions. This concern has motivated the development of various storage systems, such as batteries [1,2] and supercapacitors [3,4]. Among these energy storage technologies, batteries appear to be the most promising for electrical applications such as portable electronic devices (drones, smartphones, pacemakers, etc.), mainly due to their high-energy storage capacity, miniaturized size, long life, low weight, and low cost [5,6,7,8,9]. Today, of all secondary batteries, lithium-ion batteries (LIBs) are the most widely used technology and have attracted a great deal of attention from the scientific community. This is due to their excellent performance, such as compactness, high-power, high-energy density, and high cycling capacity. LIBs are the most widely used secondary batteries as energy sources in portable technologies and electric vehicles [5,6,10,11] and as energy storage systems in solar and wind power plants [12]. Research into overcoming the difficulties associated with the limitations of LIBs and improving their performance is a hot topic in materials science. The main limitations of LIBs are under-utilization, electrode material degradation due to cycling, capacity reduction, reduced lifetime, and the risk of thermal runaway [13,14]. Some limitations have been overcome, such as under-utilization, reduced lifetime, and the risk of thermal runaway. However, others are still being investigated. In fact, LIBs are being used in various applications, which is making them more popular, but even if their lifetime starts to become acceptable, they still need further improvement. Recent progress has focused on the preparation of innovative anode materials with optimized properties, which help to maintain the battery’s capacity during its cycle. It is well known that electrode materials play a major role in determining battery efficiency. The main drawback of anode materials is that their volume expands and contracts during the battery cycle, which can lead to cracking of the anode materials. This can lead to a drastic reduction in capacity, as well as the battery exploding due to overcharging [15,16].

Various strategies have been reported to overcome the reduction in anode volume expansion, including a better choice of material composition and/or architecture. In terms of material composition, TiO_2_ polymorphs mainly anatase, rutile, brookite, and bronze have been used for LIB applications and have been shown to exhibit a small volume change, less than 4%, during Li^+^ lithiation/delithiation (insertion/extraction) [17,18]. TiO_2_ polymorphs have other advantages that make them an ideal candidate for LIB applications, such as high mechanical and chemical stability, environmental friendliness, low cost, high cyclability, and a relatively high theoretical capacity of 335 mAh g^−1^ (anatase phase). Compared to the graphite anode in commercial LIBs, TiO_2_ (B) offers a similar theoretical capacity but a flatter, higher operating discharge voltage (>1.7 V compared to Li/Li^+^), which allows safer operating conditions [19,20]. The theoretical capacity of TiO_2_ is never reached due to various phenomena occurring during the LIB cycle. In fact, in most of the results reported in the literature, the capacity drops drastically after the first few cycles, and this is due both to the formation of a solid electrolyte interphase (SEI) layer on the surface of the anode materials resulting from the reaction of the electrolyte with the anode surface and to the disintegration of the anode material due to the stress induced by its volume variation during LIB cycles. To maintain a higher capacity and ensure longer reversible charge and discharge cycles, it is important to solve these problems. Identifying the morphology and architecture of materials that could accommodate the variation in anode volume and, consequently, reduce the stresses experienced by the materials during battery cycling, is of paramount importance in solving this problem. The aggregation of nanoparticles has proven to be a promising approach for obtaining materials with the desired architecture to attenuate the volume variation during LIB charge/discharge cycles [21]. Assemblies of nanoparticles of different sizes offer numerous possibilities for tailoring the porosity of agglomerated materials, in terms of pore size and morphology [22,23]. In addition, nanoparticle assemblies give rise to several cases of pore connectivity [24]. This type of material architecture is more flexible, making it possible to combine several material properties, even if they are of a conflicting nature [22]. For LIB application, the porous materials and particularly the aggregate of nanoparticles offer the possibility to accommodate the structural stress of the anode material, induced by lithium insertion, which improves the stability of LIB recycling [23,25]. In addition, the agglomerated porous material offers a high interface surface area in contact with the electrolyte, which greatly reduces the diffusion pathways of Li^+^ ions into the material lattice [23,24,25]. 

In the present work, TiO_2_ materials with micro- and nano-structures made by TiO_2_ nanoparticles of different morphologies, as building unit, have been prepared using the hydrothermal method. Furthermore, the capacity degradation with the number of charge/discharge cycles was discussed in terms of the evolution of the morphology of the TiO_2_ nanoparticles and the properties of their assembly in terms of pore size and connectivity with the LIB cycles, in light of the LIB literature and the present results. Considering the specific surface area, the role of the TiO_2_ crystalline phase in this degradation was also discussed. TiO_2_ materials have been used as a model system to explore the underlying mechanisms of capacity degradation with the first charge and discharge cycles.

## 2. Materials and Methods

### 2.1. Synthesis of TiO_2_ Nanoparticles and Aggregates

TiO_2_ nanoparticles in the form of urchins, nanotubes, and nanobelts were synthesized by the hydrothermal method. TiO_2_ aggregates were used as precursors and were prepared in accordance with previously published protocols [26]. To synthesize the nanoparticles, 0.5 g of TiO_2_ aggregate precursor powder was placed in a 23 mL Teflon-lined autoclave. It was then filled with 10 M NaOH to 80% of its volume. Depending on the desired morphology, the synthesis temperature was maintained at different temperatures of 100, 150, and 220 °C with a heating rate of 2.5 °C/min and synthesis times of 360, 180, and 15 min.

The nanoparticle powders thus obtained were then subjected to washing and annealing protocols to obtain sodium titanate in the final stage. The latter product was rinsed several times, first with a dilute solution of HCl to reach a pH of 1, and then with distilled water to reach a pH of 7. At the final stage of synthesis, the powder was dried overnight in an oven at a temperature of 80 °C, then annealed in air at a temperature of 500 °C for 30 min at a heating rate of 5 °C/min. All chemicals used were of analytical grade and were used as received. The water used was filtered using the Milli Q system (Millipore (Burlington, MA, USA), resistivity 18.2 MΩ.cm).

Four powders were prepared with different morphologies depending on the synthesis temperature and time. The powders are named TiO_2_-NU-100 °C (nanourchin-like nanoparticles prepared at a synthesis temperature and time of 100 °C and 360 min, respectively), TiO_2_-NU-150 °C (nanourchin-like nanoparticles prepared at a synthesis temperature and time of 150 °C and 360 min, respectively), TiO_2_-NT-200 °C (nanotube-like nanoparticles prepared at a synthesis temperature of 200 °C and a synthesis time of 15 min) and TiO_2_-NT-200 °C (nanobelt-like nanoparticles prepared at a synthesis temperature of 200 °C and a synthesis time of 360 min).

### 2.2. Characterization of Prepared TiO_2_ Nanoparticles and Aggregates

Morphological studies of TiO_2_ nanoparticles and aggregates were carried out using high-resolution Zeiss Ultra 55 field-emission scanning electron microscopes (FE-SEM) under the conditions of an accelerating voltage of 10 kV. 

The identification of the TiO_2_ crystal structure was carried out with an X-ray diffractometer (Siemens D5000 XRD unit, Munich, Germany) in the range of 2θ between 20° and 80° in progressive steps of 0.07° s^−1^, at an accelerating voltage of 40 KV and a current of 40 mA using a Cu Kα radiation source with λ = 1.5406 Å. The average TiO_2_ crystallite size was determined using the Scherer formula: (D = 0.9λ/Bcosθ) using the half-height width of the intense peak corresponding to the crystallographic plane (020).

Nitrogen adsorption–desorption isotherms were measured using a BelSorp Max apparatus at liquid nitrogen temperature. The samples were degassed at 120 °C for 10 h before measurement. The specific surface area (SBET) was estimated using the Brunauer–Emmett–Teller (BET) method in the P/P° range between 0.05 and 0.25. Pore size distribution was assessed from isothermal desorption using the non-local density functional theory (NLDFT) method (cylindrical pores; oxidised materials). Total pore volume was estimated from the amount of N_2_ adsorbed up to P/P° = 0.98.

The chemical composition of all samples was identified by FE-SEM equipped with an energy dispersive spectrometry (EDS) system from Princeton Gamme-Tech PGT, Princeton, NJ, USA. 

Electrochemical tests were carried out in Teflon Swagelok half-cells using TiO_2_-based powder as the working electrode and Li metal foil (Sigma Aldrich, St. Louis, MI, USA) as both the reference and counter electrode. Battery grade electrolyte was purchased from (Solvionic, Toulouse, France) to prepare the cells with the following composition LiPF6 1M ethylene carbonate, propylene carbonate, and dimethyl carbonate (1:1:1, v/v/v) with 1% by weight vinylene carbonate. The TiO_2_-based working electrode powder was prepared using a mortar from a mixture of prepared TiO_2_ active material (80 wt%), 7 wt% mesoporous carbon, 7 wt% graphite powder, and 6 wt% poly(tetrafluoroethylene) (PTFE). The prepared homogeneous mixture was uniformly pressed onto a stainless-steel sheet at a pressure of 125 bar. The prepared electrode was oven-dried overnight at a temperature of 80 °C. A glass microfibre filter (Grade GF/D (What-man)) with a thickness of 0.67 mm and a pore size of 2.7 μm (GmF) was used as a separator. The Swagelok cells were assembled in an MBraun glove box under the following conditions: H_2_O < 1 ppm and O_2_ < 1 ppm. The assembled batteries were galvanostatically cycled in the voltage range of 3 to 1.0 V against Li/Li^+^ at a charge/discharge rate of C/10 (full charge or discharge in 10 h) using a Biologic VMP3 multi-channel potentiostat/galvanostat.

## 3. Results and Discussion

**Figure 1:**
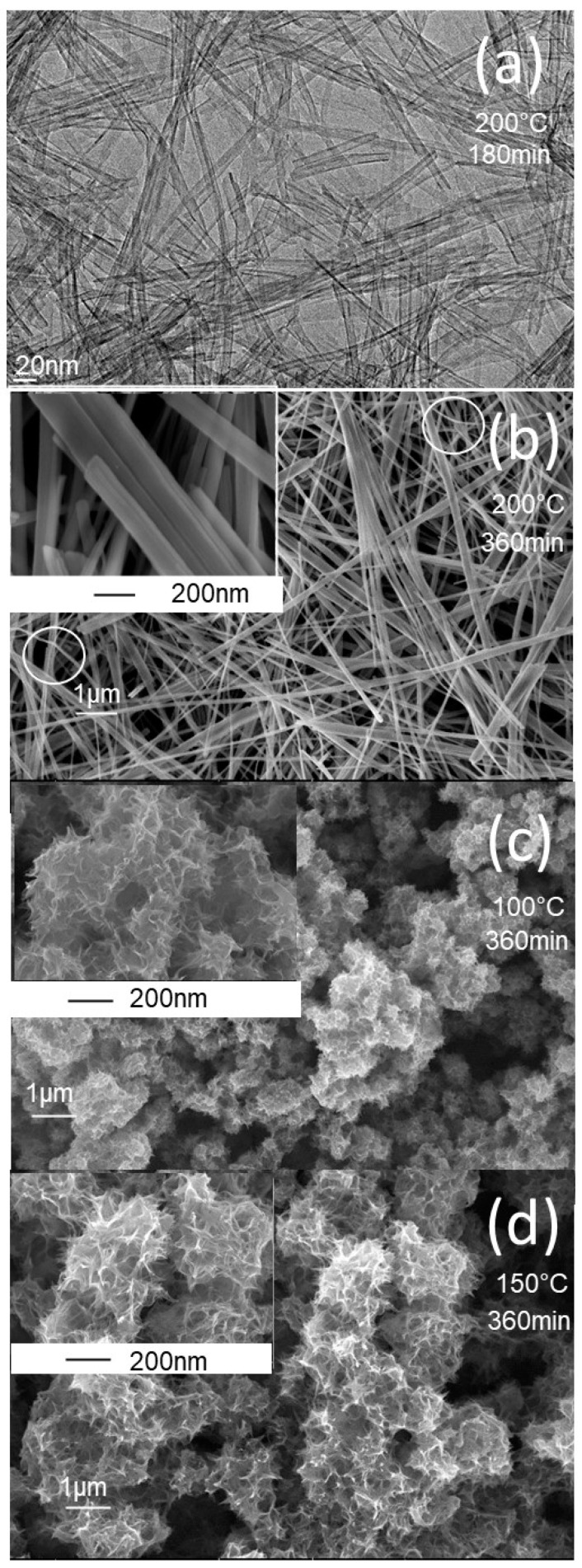
FE-SEM and TEM images of TiO_2_ powder of different morphologies prepared at different synthesis times and temperatures. (**a**) TEM image of TiO_2_-NT-200 °C powder prepared at synthesis temperature of 200 °C, over 180 min, (**b**) FE-SEM image of TiO_2_-NB-200 °C powder prepared at synthesis temperature of 200 °C over 360 min, (**c**) FE-SEM image of TIO_2_-NU-100 °C powder prepared at synthesis temperature of 100 °C, over 360 min and (**d**) FE-SEM image of TiO_2_-NU-150 °C powder prepared at synthesis temperature of 150 °C, over 360 min. The inserts are the corresponding high magnifications.

The synthesis protocols described in the experimental section yielded white powders that were characterized using a variety of techniques. The morphology of the prepared powders was characterized by FE-SEM, and the images obtained are shown in Figure 1. At a higher synthesis temperature of 200 °C (Figure 1a,b), the TiO_2_ powder exhibits a nanotube morphology at a short synthesis time of 180 min, and a nanobelt morphology at a longer synthesis time of 360 min. The insert in Figure 1b shows a bundle of several nanobelts stacked along their longitudinal axis. It can also be seen that the nanobelts prepared have a homogeneous thickness of around 10 nm, a diameter ranging from 50 to 100 nm, and a length of more than 10 μm. At higher magnifications, the nanobelts have a smooth surface with no contamination. In addition, the regions shown in Figure 1b display curved nanobelts, illustrating their high elasticity. At the synthesis temperature of 100 °C, the prepared powder has a sea-urchin-like morphology, with stretched sheets connected to form a randomly connected network (Figure 1c). At the synthesis temperature of 150 °C, the morphology is like that obtained at a temperature of 150 °C, but with more coiled sheets (Figure 1d).

**Figure 2:**
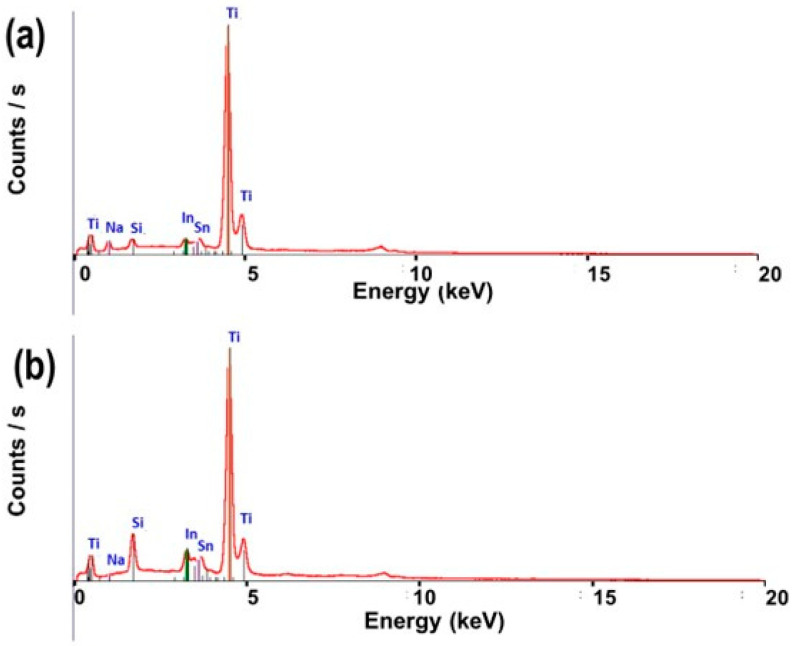
EDS spectrum images of prepared TiO_2_ powders (**a**) just after synthesis and (**b**) after washing and annealing processes (Si, In, and Sn peaks are assigned to the ITO substrate).

The EDS spectrometry was used to analyze the chemical composition of prepared TiO_2_ powders, just after synthesis (Figure 2a) and after the washing and annealing steps (Figure 2b). The EDS spectra show the peak corresponding to Na just after synthesis Figure 2a, whereas after the washing and annealing steps, the Na peak is completely absent (Figure 2b), which indicates the complete exchange of Na^+^ ions by H^+^ during the washing step. 

**Figure 3:**
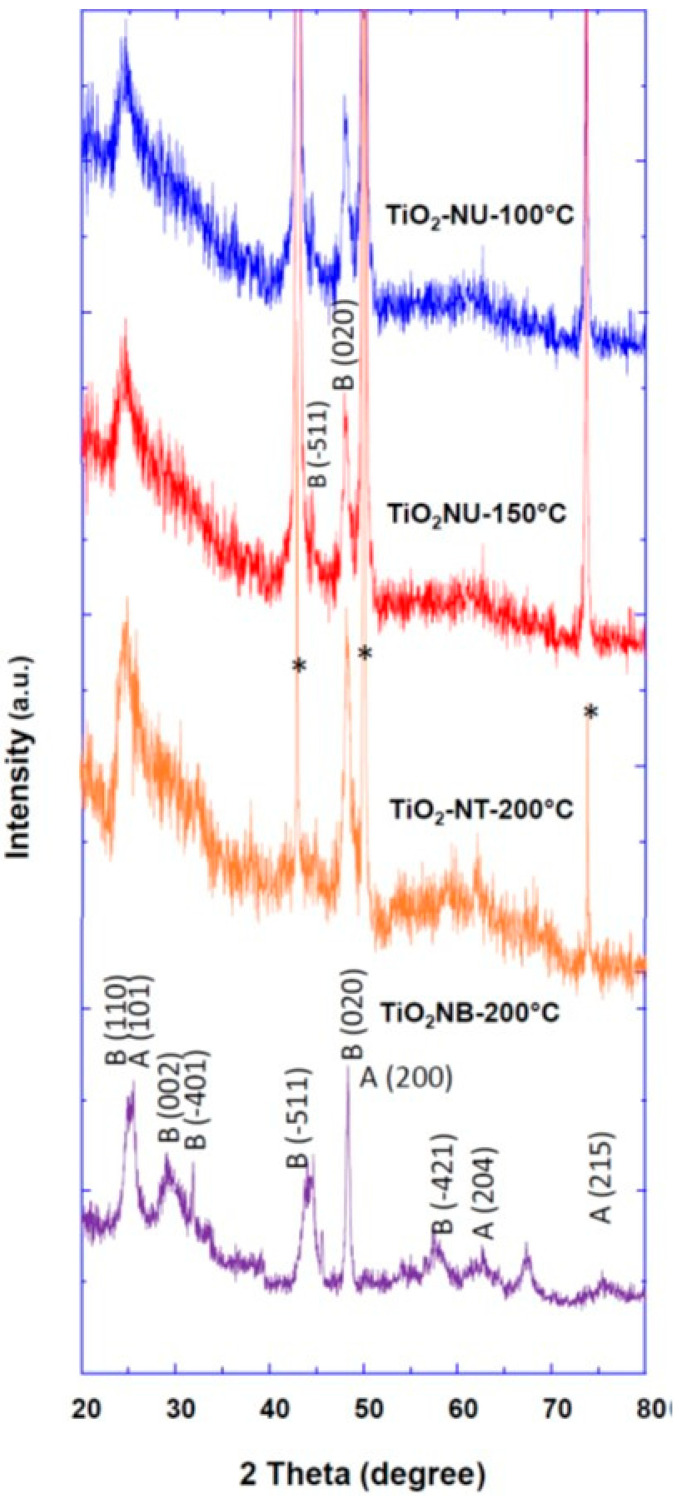
XRD pattern of TiO_2_ powders with different morphologies prepared at different synthesis temperatures as indicated. The peaks with stars correspond to the substrate.

The crystalline structure and phase of prepared TiO_2_ powder with different morphologies were investigated by the XRD method, and the obtained patterns are depicted in Figure 3. In the case of TiO_2_ nanourchin and nanotube morphologies (TiO_2_-NT-200 °C, TiO_2_-NU-150 °C and TiO_2_-NU-100 °C), well-pronounced peaks were observed and were assigned to (−511) and (020) crystallographic planes of pure TiO_2_ (B) phase (JCPDS No. 35-008) (Figure 3). In the case of TiO_2_ nanobelt morphology (TiO_2_-NB-200 °C), the well-resolved XRD peaks were attributed to a mixture of anatase (JCPDS 83-2243) and brookite (JCPDS 29-1360) phases (Figure 3).

**Figure 4:**
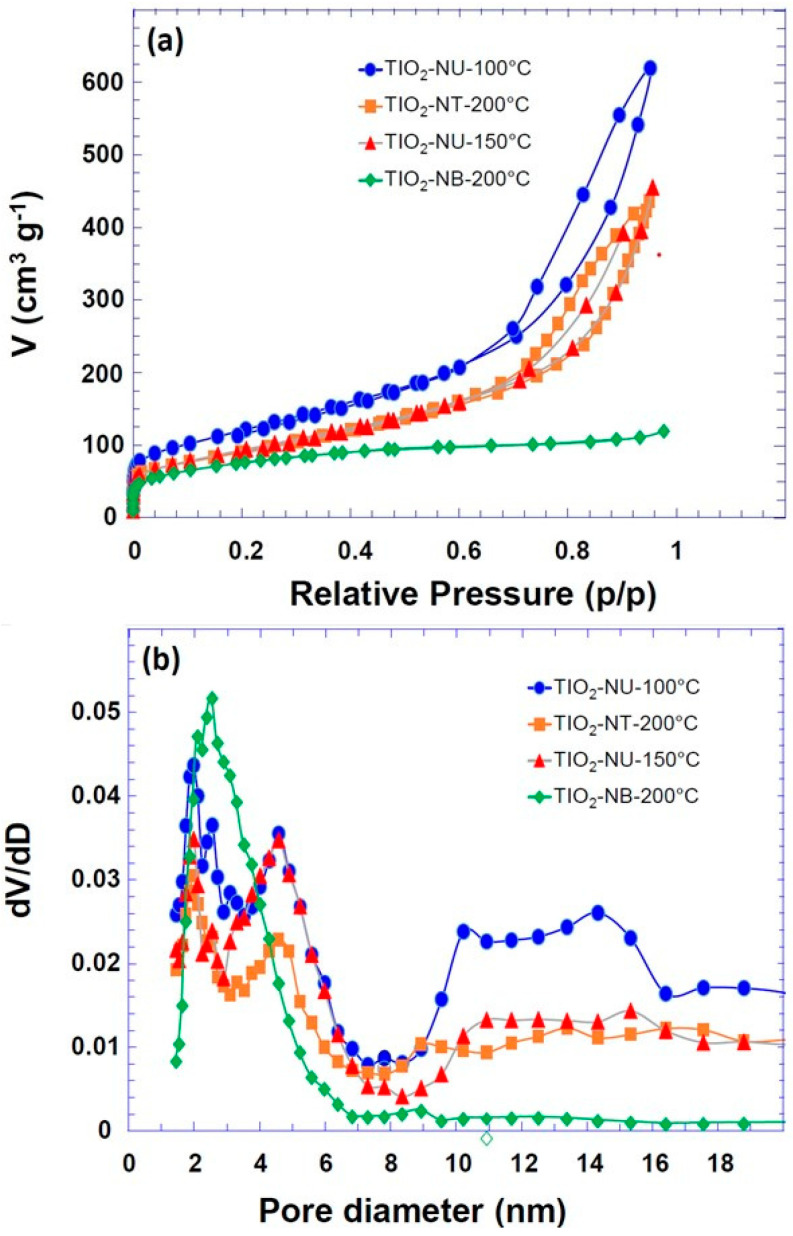
(**a**): N_2_ physisorption isotherm of TiO_2_ powders with different morphologies as indicated; (**b**): the corresponding pore size distribution (NLDFT).

The properties of prepared TiO_2_ powders in terms of specific surface area, and the average pore size, were evaluated by analyzing the nitrogen adsorption–desorption isotherms. From the isotherm curves (Figure 4), the specific surface areas (BET model) were calculated to be 270 m^2^g^−1^, 329 m^2^g^−1^, 434 m^2^g^−1^, and 335 m^2^g^−1^, for, respectively, the TiO_2_ powder morphology of nanobelt (TiO_2_-NB-200 °C), nanotube (TiO_2_-NT-200 °C), nanourchin (TiO_2_-NU-100 °C), and (TiO_2_-NU-150 °C). For most of the samples a multiscale porosity is observed (Figure 4a,b): the smaller pores (2.5–3 nm) are likely due to the intrinsic porosity of the particles (porosity of the nanotube, nanobelt, or nanosheet), while the porosity leading to the second maximum in the pore size distribution (about 5 nm) (Figure 4a,b) is probably related to pores resulting from the aggregation of the primary particles. Lastly, the larger pores (between 10 and 20 nm) could result from the flexible porosity formed between particles that are not chemically linked. It is worth noting that the porosity of TiO_2_-NU-150 °C is like that of TiO_2_-NT-200 °C, which is consistent with the fact that the nanotube particles are obtained by nanosheets enrolling as reported previously [26]. For the TiO_2_-NB-200 °C sample, the isotherm (Figure 4a) is completely reversible, which is characteristic of adsorption in narrow mesoporous pores smaller than 4 nm, whereas for the other samples, the isotherms show the existence of hysteresis associated with adsorption in pores larger than 4 nm. Furthermore, the appearance of hysteresis is thought to be related to capillary condensation in large pore channels and may also be related to pore connectivity [27].

It is commonly accepted that the large surface area enhances the material contact surface with the electrolyte, and the large pore size favors fast diffusion and transfers toward the material surface [5,24,25]. These characteristics favor the improvement of LIB rate capability. 

**Figure 5:**
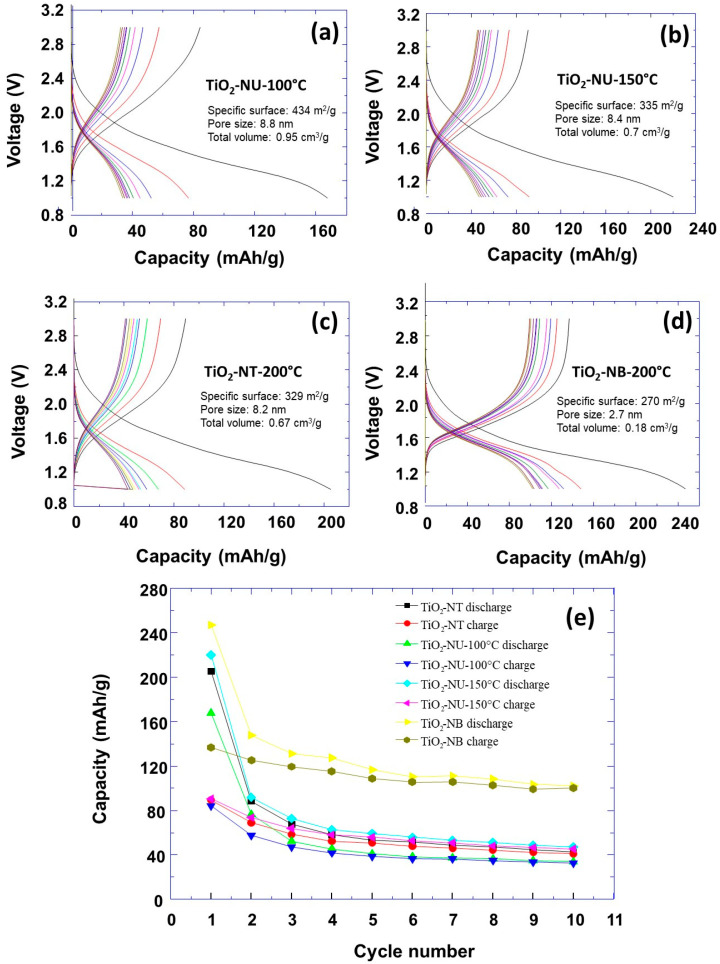
The potential–capacity curves corresponding to discharge/charge cycles of the first 10 cycles for different TiO_2_ powder morphologies (**a**–**d**). The curves of specific capacity versus the number of charge/discharge cycles for different TiO_2_ powder morphologies (**e**).

The electrochemical characterizations of prepared TiO_2_ powders with different morphologies were performed. The obtained discharging/charging curves at a current rate of C/10 are shown in Figure 5.

It can be observed that the specific capacity decreases as a function of the number of discharging/charging cycles (Figure 5e). At the first initial discharging process, the highest capacity of about 250 mAh/g was observed for the nanobelt morphology. For the other morphologies, this initial capacity was about 210 mAh/g, 170 mAh/g, and 220 mAh/g for, respectively, the TiO_2_ powder morphologies of nanotube (TiO_2_-NT-200 °C) and nanourchin (TiO_2_-NU-100 °C and TiO_2_-NU-150 °C). It is important to note that the specific capacities corresponding to the different morphologies are lower than the theoretical capacity of TiO_2_, which is approximately 336 mAh/g. One could expect that higher capacities are associated with materials with a higher specific surface, which provide a higher contact surface area with the electrolyte. For example, it can be observed that TiO_2_ powders of nanotube and nanourchin-150 morphologies show similar specific capacity, which could be explained by their similar specific surface area. Moreover, TiO_2_ powder with nanourchin morphology shows more enrolled nanosheets (TiO_2_-NU-150 °C) which resembles that of the nanotube morphology. However, the TiO_2_-NU-100 °C powder has a higher specific surface than nanourchin-150 (434 vs. 335 m^2^/g) and should, normally, exhibit a higher capacity. The electrochemical measurements show a lower capacity, which is very surprising, if only the specific surface parameter is considered. After the first discharging step, the specific capacity decreases very fast for all prepared TiO_2_ powder morphologies, and it reaches a plateau after a few numbers of discharging/charging cycles. To understand this behavior, another phenomenon of individual nanoparticles should be discussed to explain the observed variation of the specific capacity versus the number of discharging/charging cycles. It was reported that the anode material expansion and shrinkage during the lithiation/dilithiation induces the formation of cracks and the initial TiO_2_ particles disintegration into small nanoparticles. This in turn provokes the electric disconnection between the current collector and the anode materials. This lowers the LIB cycling stability and specific capacity [2]. Furthermore, the observed irreversible capacity during the first cycle could be explained mainly by the formation of the passivating layer named solid electrolyte interphase layer (SEI) on the electrode surface because of the electrolyte reduction [28,29,30], and the trapping of the inserted lithium in the crystal lattice defects or on the electrode surface sites [31]. This explains the lower capacity observed than the theoretical calculated capacity which is equal to 335 mAh g^−1^ [19,32,33].

Hereafter, we will discuss how the control over the TiO_2_ powder morphology may be used to improve the Li-ion batteries’ performance. It is well accepted that the pore properties (size and connectivity) and the specific surface depend on the prepared TiO_2_ powder morphology, and it plays a crucial role in the optimization of the Li-ion batteries’ specific capacity [34]. It has been reported that TiO_2_-based electrode materials with smaller particles and/or greater porosity contribute to improved electrochemical performance [35,36,37,38,39].

**Figure 6:**
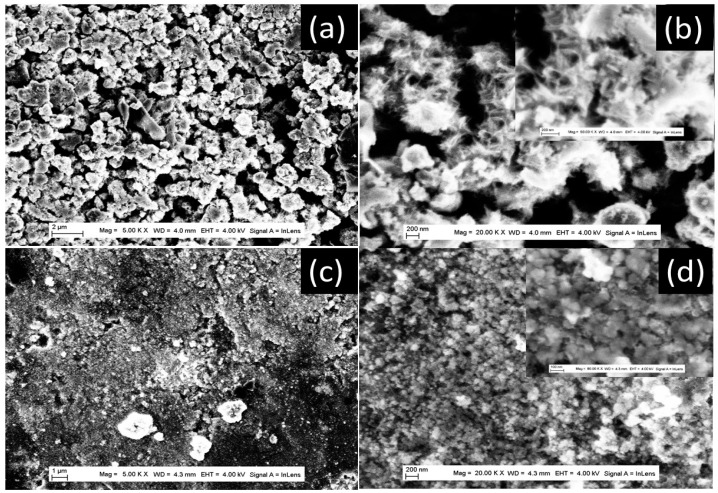
FE-SEM images of TiO_2_ powder with nanourchin 100 morphology (**a**,**b**) different magnifications of the anode materials after preparation (**c**,**d**) different magnifications de anode materials after 10 discharging/charging cycles. The inserts are the corresponding high magnification.

**Figure 7:**
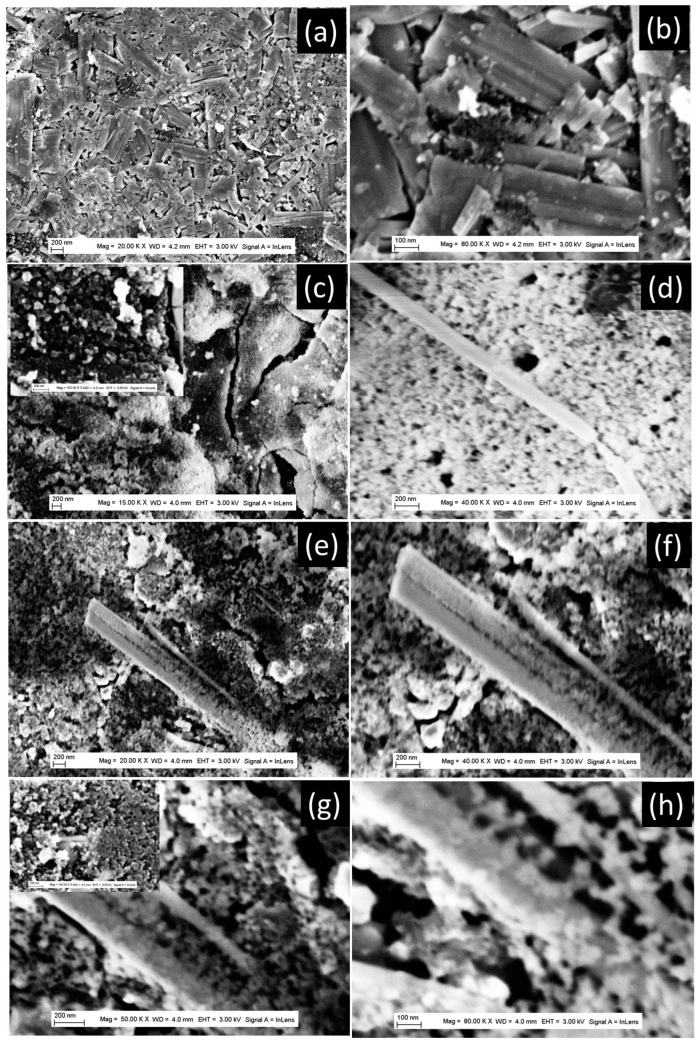
FE-SEM images of TiO_2_ powder with nanobelt morphology; (**a**,**b**) different magnifications of the anode materials after preparation; (**c**–**h**) different magnifications of de anode materials after 10 discharging/charging cycles. The inserts are the corresponding high or low magnification.

**Figure 8:**
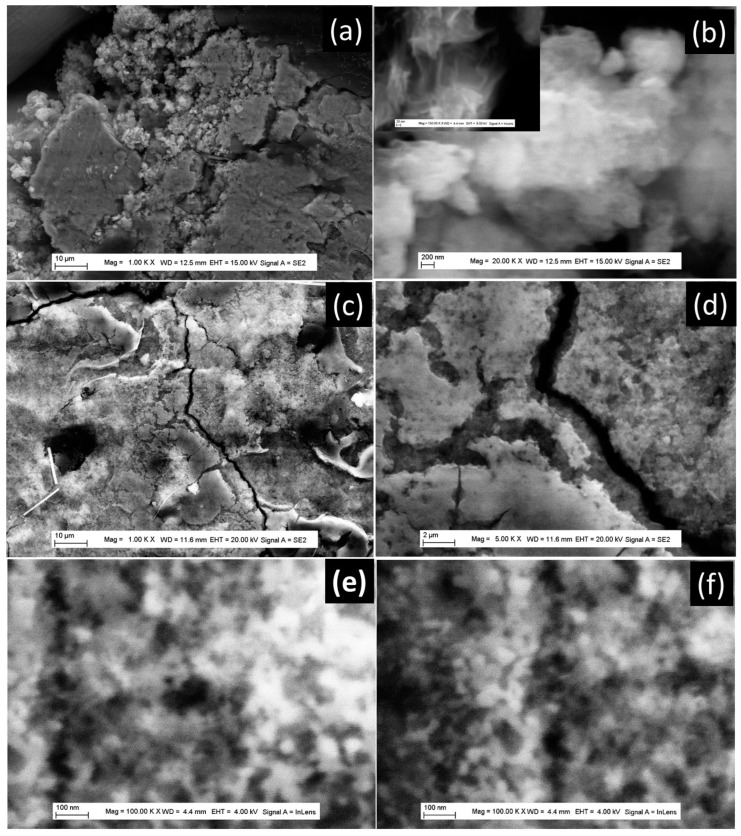
FE-SEM images of TiO_2_ powder with nanotube morphology; (**a**,**b**) different magnifications of the anode materials after preparation; (**c**–**f**) different magnifications of de anode materials after 10th discharging/charging cycles. The inserts are the corresponding high magnification.

To understand how these parameters are behind the observed decrease in the specific capacity, versus the discharging/charging cycles, we must investigate the evolution of the TiO_2_ powder morphology during the cycling process. To check this point, the FEGSEM characterization was performed just after the preparation of the anode for battery testing and after 10 discharging/charging cycles. The results are presented in Figure 6, Figure 7 and Figure 8, for prepared TiO_2_ powders with different morphologies. The TiO_2_-NU-100 °C powder, with stretched nanosheets, starts to collapse during the preparation of the anode electrode (Figure 6a,b), inducing a decrease in the anode-specific surface. After the 10th discharging cycle, it can be observed that the nanosheets of nanourchin morphology mostly collapsed, to form aggregates of around 100 nm diameter (Figure 6c,d). The observed peculiarity with the TiO_2_-NU-100 °C powder, in terms of low specific capacity (Figure 5), despite that it is characterized by the highest specific surface just after synthesis, could be explained by the fact that the stretched sheet forming nanourchin morphology is easy to collapse, during the battery’s fabrication process. After their preparation, the nanourchin morphology evolves to a denser structure with a lower specific surface than that of nanobelt morphology. Similar nanosheets collapse behavior was previously observed with TiO_2_ powders of nanourchin morphology by Tian-Hui et al. [40].

Similar behavior was observed with TiO_2_-NB-200 °C powder, in terms of a strong decrease in the LIB-specific capacity between the first and the 10th discharging cycle (Figure 5). To understand this behavior in the case of nanobelt morphology, we closely analyzed the FE-SEM characterization before battery testing, and after the 10th discharging cycle (Figure 8). Just after the preparation of the anode, the TiO_2_ powder keeps its nanobelt morphology as it can be identified in the FE-SEM pattern of Figure 7a,b. After the 10th cycle, only the aggregates with diameters ranging from 50 nm to 200 nm could be observed, in addition to a few nanobelts (Figure 7c–h). Further analysis of the FE-SEM images (Figure 7e–h) shows a belt-shaped nanoparticle that is in the process of disintegrating with a coexistence of a part of the particle that is transformed into particle aggregates and another that is not yet. This clearly shows that the aggregates observed are the result of the disintegration of the belt-shaped nanoparticles under the effect of the stress generated by the lithium-ion insertion/extraction process during the charge and discharge cycles. (Figure 7e–h). It is well known that lithium storage capacity in the anode material induces its expansion during the lithium insertion, which can provoke mechanical fracture in individual nanobelts, and its disintegration into aggregates.

With TiO_2_-NT-200 °C powder, aggregates are formed during the battery’s fabrication process (Figure 8a,b). At high magnification in Figure 8b and the insert, TiO_2_ nanotubes could be observed. After the 10th cycle, cracks are formed (Figure 8c,d), and aggregates of nanotubes could be observed with a diameter ranging from 20 nm to 50 nm (Figure 8e,f). Regarding TiO_2_-NU-150 °C powder, it collapses during the battery’s fabrication process but with less intensity than in the case of TiO_2_-NU-100 °C. This is due to its nanosheet enrolled structure, which provides more resistance to the change of the powder’s morphology during the fabrication process. TiO_2_ nanotubes and their aggregation led to a reduction in surface area, which explains its lower capacity compared to that of nanobelt powder. 

It is well known that nanoparticle aggregates are usually porous materials characterized by pore size, size distribution, connectivity, and specific surface. Furthermore, it is well accepted that these parameters affect the Li-ion diffusion within the LIB electrode and are also behind the volume accommodation during the insertion/extraction cycle of Li-ion [2,23,25]. The obtained results are very surprising if we consider only the geometrical model in which the reduction in the pore size induces the enhancement of the specific surface, and in turn, the specific capacity as previously observed by Lin et al. [21].

In addition, the pore’s connectivity should play an important role in the optimization of energy storage of porous electrodes [41]. However, during the first discharging cycle, the TiO_2_ powders keep different morphologies and probably different connectivity, which could also explain the observed difference in specific capacities for all the TiO_2_ powders, in addition to their specific surface. The small variation in the plateau capacity observed in Figure 5e after the 5th cycle can be explained by the reversible insertion/extraction of lithium ions during the charge/discharge process of the LIB. This can be attributed to the anode material architecture in terms of pore properties (size, shape, and connectivity) which successfully mitigates the effect of anode volume variation on battery capacity during charge/discharge cycles.

The previous results, in terms of particles disintegration, were confirmed by XRD experiments (Table 1), which show that the crystallite size decreases between the 1st and the 10th cycle. This confirms that during the discharging/charging cycles, large particles were disintegrated into small ones. Furthermore, by comparing the crystallite size after the 10th cycle, we note that the nanobelt morphology shows small crystallite, which should have a high specific surface, explaining the corresponding high specific capacity. This result is in good agreement with the literature [35,36,37,38,39]. Furthermore, it can be observed from Table 1 that the TiO_2_-NB-200 °C sample with TiO_2_ nanobelt morphology has a higher retention capacity of 44% compared to other samples with approximately the same retention capacity. This performance could be explained by the small crystallite size of the TiO_2_ aggregates, which better mitigates the volume variation and in turn reduces the TiO_2_ disintegration. In addition, the other morphologies have approximately the same crystallite size, which explains their closer specific capacity and retention of approximately 22%. These results also confirm that the size of crystallite after the same number of LIB charge–discharge cycles depends on the properties of the powders used as active electrode materials, such as morphology, size, crystallinity, and the nature of the phases.

The high performance of the TiO_2_-NB-200 °C sample compared to other samples in the plateau region observed in Figure 5 can also be explained by the influence of the crystalline phase structure of the TiO_2_ material. In fact, the electrochemical performance of TiO_2_ is closely related to its various properties such as size, morphology, specific surface area, and crystalline structure phase. The insertion/extraction of Li^+^ ions into the TiO_2_ structure can be described by the following Equation (1):

xLi^+^ + TiO_2_ + xe → Li_x_TiO_2_; (1)

Depending on the crystalline structure and size of the TiO_2_ material [42], the value of x, which represents the lithium-ion insertion capacity, is between 0 and 1. In general, reducing the size significantly improves the lithium insertion capacity in the TiO_2_ structure, regardless of its crystalline structure [42]. In addition, the crystalline structure also influences the Li-ion insertion capacity, the bronze and brookite phases of TiO_2_ have more open structures, which gives them better Li-ion insertion capacity [42]. The crystalline structure of TiO_2_(B) belongs to the monoclinic (C2/m) crystal system, and its crystal lattice is formed by an assembly of TiO_6_ octahedra sharing edges and corners, revealing an open channel located between the axial oxygen atoms. The open crystalline structure of TiO_2_(B) allows rapid diffusion of Li^+^ and, therefore, rapid charging of the LIB and its stability tends to improve its lifetime. In addition, TiO_2_(B) has the lowest density (3.73 g/cm^3^) and its structure is more porous than that of the anatase and rutile phases, which allows it to better limit volume changes during the Li^+^ insertion and extraction processes [43,44]. It is generally accepted that the TiO_2_(B) phase has higher performance as an anode material compared to other TiO_2_ phases. Considering the previous discussion, it can be concluded that the TiO_2_NB-200 °C sample will have the lowest capacity since the proportion of the bronze phase is lower, as it is formed by a mixture of bronze and anatase phases. Since the results obtained contradict this prediction based solely on the effect of the TiO_2_ phase, another effect must be considered. Indeed, if we consider the crystallite size, which is smaller in the case of the TiO_2_NB-200 °C sample, and which must have a large specific surface area, we can explain the better performance of this sample in terms of the high capacity compared to other samples. It can also be concluded that the effect of the crystallite size on the LIB capacity outweighs the effect of the phase in controlling the performance of the LIB in terms of high capacity.

LIB performance also depends on the electronic and ionic conductivity of the anode material. Spinner et al. [45] reported that conductivity and structure have a strong influence on the reversibility, rate capability, capacity, and capacity retention of nickel oxide (NiO) anodes for LIBs. They show that the cycling reversibility of nickel oxide as an anode material during the charging/discharging processes was greatly improved after the addition of carbon, which increases its conductivity. Carbon-based powders such as carbon nanotubes, graphite, etc., are commonly used to improve the conductivity of metal oxide-based anode materials. The carbon has been shown to improve charge conductivity, Li-ion diffusion, attenuation of volume changes during the charge/discharge process and prevent aggregation of active materials by forming the passivating layer [46,47,48]. The addition of carbon improves conductivity by allowing inactive anode particles resulting from the degradation of the anode materials to retain their activity after many cycles.

In the present work, all the TiO_2_ anode powders were prepared by carbon addition in the same way and proportion; therefore, the better capacity retention of 44% and the cyclability of TiO_2_NB-200 °C cannot be explained by the addition of carbon. However, if the crystallite size is considered, it can be observed that TiO_2_NB-200 °C has the smaller crystallite, which could improve its electrical contact between the active materials and the conductive carbon used to prepare the anode material, resulting in the improved conductivity of TiO_2_NB-200 °C. This may also help to explain the high capacity of TiO_2_NB-200 °C compared to other samples.

## 4. Conclusions

It has been shown that the low-capacity retention observed, ranging from 22% to 44% after 10 LIB discharge/charge cycles, is due to the evolution of the morphology during the battery preparation process and its first discharge/charge cycles. Except in the case of nanobelt morphology, there is only a small influence on the capacity of the other morphologies after the 5th cycle. During battery cycling, there is a decrease in the size of the TiO_2_ crystallites because of the disintegration of the primary particles, and the size of these crystallites is dependent on the initial morphology of the powders. These results, which demonstrate the evolution of morphology during battery cycling, also highlight the role of pore size and connectivity in the variation of LIB capacity. It was observed that the TiO_2_-NB-200 °C sample exhibited the highest capacity of 250 mAh/g and a retention of 44% after 10 cycles, which was explained by the small size of the TiO_2_ crystallites compared to the other samples during LIB cycling. It can also be concluded that the effect of the crystallite size on the LIB capacity outweighs the effect of the phase in controlling the performance of the LIB in terms of the high capacity of the TiO_2_-NB-200 °C sample. In addition, the plateau observed in the variation of the battery capacity as a function of the number of discharge/charge cycles was attributed to the enhanced reversibility of the Li-ion insertion/extraction process favored by the open structure of the TiO_2_(B) phase and the porous architecture of the TiO_2_ aggregates. Controlling the size and the phase of the TiO_2_ crystallites, as well as the characteristics of the pores in terms of size and connectivity, proved to be a key issue to better mitigate the variation in anode volume to improve the performance of LIBs in terms of improved retention and cyclability.

These results will make a significant contribution to our understanding of the cyclability of LIBs and the variation of their specific capacity during initial discharge/charge cycles. They will provide new insights into the design of the best electrode material architectures to improve LIB performance.

## Figures and Tables

**Table 1:** Characteristics of TiO_2_ powders after preparation of the anode electrode and after 10 discharging/charging cycles.

	TiO_2_NT-200 °C	TiO_2_NU-100 °C	TiO_2_NU-150 °C	TiO_2_NB-200 °C
Specific surface	329 m^2^g^−1^	434 m^2^g^−1^	335 m^2^g^−1^	270 m^2^g^−1^
Initial specific capacity	210 mAh/g	170 mAh/g	220 mAh/g	250 mAh/g
Specific capacity after 5 cycles	60 mAh/g	45 mAh/g	65 mAh/g	120 mAh/g
Specific capacity after 10 cycles	45 mAh/g	40 mAh/g	50 mAh/g	110 mAh/g
Specific capacity retention after 10 cycles	21%	23%	22.72%	44%
Crystallite size after synthesis	198.8 nm	79.3 nm	82.6 nm	219.7 nm
Crystallite size after 10 cycles	66.1 nm	61.4 nm	60.2 nm	45.3 nm

## Data Availability

The data presented in this study are available on request from the corresponding author.
